# The Human Cathelicidin LL-37 Has Antiviral Activity against Respiratory Syncytial Virus

**DOI:** 10.1371/journal.pone.0073659

**Published:** 2013-08-30

**Authors:** Silke M. Currie, Emily Gwyer Findlay, Brian J. McHugh, Annie Mackellar, Tian Man, Derek Macmillan, Hongwei Wang, Paul M. Fitch, Jürgen Schwarze, Donald J. Davidson

**Affiliations:** 1 MRC Centre for Inflammation Research, Queen's Medical Research Institute, The University of Edinburgh, Edinburgh, Scotland, United Kingdom; 2 Department of Respiratory Medicine, Affiliated Nanjing Children's Hospital of Nanjing Medical University, Nanjing, PR China; 3 Department of Chemistry, University College London, London, United Kingdom; 4 Center for Translational Medicine, Medical School of Nanjing University, Nanjing, PR China; University of Iowa, United States of America

## Abstract

Respiratory syncytial virus is a leading cause of lower respiratory tract illness among infants, the elderly and immunocompromised individuals. Currently, there is no effective vaccine or disease modifying treatment available and novel interventions are urgently required. Cathelicidins are cationic host defence peptides expressed in the inflamed lung, with key roles in innate host defence against infection. We demonstrate that the human cathelicidin LL-37 has effective antiviral activity against RSV *in vitro*, retained by a truncated central peptide fragment. LL-37 prevented virus-induced cell death in epithelial cultures, significantly inhibited the production of new infectious particles and diminished the spread of infection, with antiviral effects directed both against the viral particles and the epithelial cells. LL-37 may represent an important targetable component of innate host defence against RSV infection. Prophylactic modulation of LL-37 expression and/or use of synthetic analogues post-infection may represent future novel strategies against RSV infection.

## Introduction

Respiratory syncytial virus (RSV) is the major cause of viral bronchiolitis in young children worldwide, and a leading cause of morbidity and mortality in infants, elderly and immunocompromised individuals [1], [2]. In addition to major morbidity (33.8 million episodes worldwide per year), RSV bronchiolitis also leads to significant mortality in the developing world with up to 200,000 deaths annually in young children worldwide [1]. RSV infection results in inflammatory lower respiratory tract disease in about 30% of infants who become infected, and, in ∼2% of all infants, RSV bronchiolitis is potentially life-threatening and requires admission to hospital (reviewed in [3]). Although most infants in developed countries recover, these children have an increased risk of recurrent wheeze and RSV infection has been implicated in the later development of asthma [4]. Despite the prevalence of RSV bronchiolitis, susceptibility remains poorly understood, there is no vaccine available and, apart from supportive measures, there is no specific effective treatment (reviewed in [5]). A clearer understanding of the host defence factors that contribute to effective protection against RSV in the majority of those exposed to this virus should inform the development of novel prophylactic and/or therapeutic strategies.

Cationic host defence peptides (CHDP; also known as antimicrobial peptides) are key components of innate defences against infection, with both microbicidal and host defence modulatory functions (reviewed in [6]). In addition to their well described bactericidal potential, CHDP have more recently been shown to have antiviral properties (reviewed in [7]). Cathelicidins are multifunctional immunomodulatory CHDP expressed in a broad range of tissues in infection and inflammation (reviewed in [8], [9]). The sole human cathelicidin, Human Cationic Antimicrobial Peptide of 18KDa (hCAP-18), from which the active form LL-37 is generated proteolytically [10], is expressed primarily by neutrophils and epithelial cells. Airway epithelial cell expression of LL-37/hCAP18 is induced *in vitro* by RSV infection [11], and is significantly enhanced by the 1,25OH metabolite of vitamin D [12]. In addition, a recent study of children with RSV bronchiolitis demonstrated that median serum levels of hCAP-18 were significantly lower in children with RSV bronchiolitis than in those with human rhinovirus induced bronchiolitis [13]. Furthermore, RSV-infected children whose hCAP-18 levels were lower than the median were found to be more likely to be hospitalised for prolonged periods than those with hCAP-18 levels above the median. These findings suggest an antiviral role for human cathelicidin in host defence against RSV.

In order to determine the antiviral potential of LL-37 against RSV, we examined the impact of LL-37 on RSV infection *in vitro*, establishing the effect of this CHDP on viral particles and its protective impact upon human epithelial cells.

##  Materials and Methods

### Peptide synthesis

**Figure 1:**
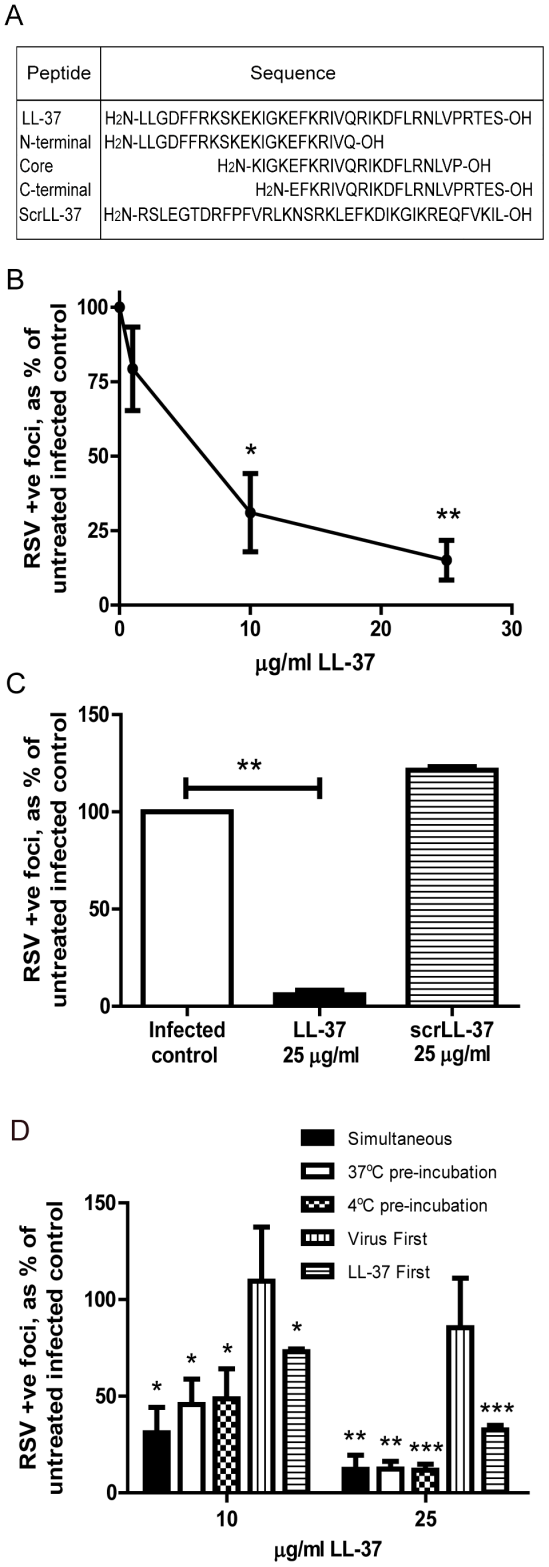
LL-37 has antiviral activity against RSV. a) Sequences of LL-37, three 22-mer peptides (representing the N-terminal, central core and C-terminal regions) and scrambled LL-37 peptide, b) HEp-2 or c) 16HBE14o^-^ cells were infected with RSV (MOI = 0.005) and simultaneously treated with LL-37 or a scrambled control LL-37 (scrLL-37) at the concentrations indicated for 2 hours. Cells were washed and incubated for 24 hours at 37°C, and then the number of infected cells was quantified. d) HEp-2 cells were exposed to LL-37 and RSV (MOI = 0.005) simultaneously (“Simultaneous” condition), for 2 hours, or exposed for 2 hours to RSV that had been preincubated with LL-37 for 1 hour (“37°C pre-incubation” and “4°C pre-incubation” conditions), or exposed to RSV for 2 hours and washed before a 1 hour incubation with LL-37 (“Virus first” condition), or treated with LL-37 for 1 hour and washed before a 2 hour infection with RSV (“LL-37 first” condition). Every treatment was followed by a wash step. After 24 hours at 37°C, the number of infected cells was quantified. b, c & d) Infected cells were quantified using an immunoplaque assay and expressed as a percentage relative to untreated, RSV infected cells. Results are shown as mean +/− SEM of n = 3-5. For statistical analysis, RSV-positive foci counts were log transformed and analysed by one way ANOVA with Dunnett Multiple Comparison Post-tests to compare treatments to untreated infected controls * p<0.05, ** p<0.01, *** p<0.001.

LL-37, TAMRA-labelled LL-37, scrambled LL-37 and truncated LL-37 partial peptides (all sequences in Figure 1a) were either purchased from Activotec (Cambridge, UK) or synthesised as described below. Peptide purity was >95% by RP-HPLC and net peptide content determined by amino acid analysis. NovaSYN TGT resin preloaded with Fmoc-Ser(tBu)-OH (0.05 mM) was transferred to an automated peptide synthesiser (ABI 433A) reaction vessel for peptide chain elongation employing 10 equivalents of each standard Fmoc-protected amino acid employing the Fastmoc™ protocol. Following chain elongation the fully protected resin-bound peptide was treated with trifluoroacetic acid: ethanedithiol: water (95: 2.5∶2.5, 4.0 ml) for 5 hours. The resin was filtered off and the filtrate was added to cold diethyl ether (40 ml), which induced precipitation. The precipitate was collected by centrifugation at 3000 rpm, 4°C for 15 min. The ether layer was then decanted and the precipitated peptide was washed once more with cold diethyl ether (40 ml). The white precipitate was then dissolved in water and purified by semi-preparative reverse phase (RP)-HPLC (gradient: 5–60% acetonitrile/45 min). Fractions containing product were identified by LC-MS, pooled and lyophilised. Lyophilised peptides were reconstituted in endotoxin free water at 5 mg/ml stock concentration and determined to be endotoxin-free using a Limulus Amebocyte Lysate Chromogenic Endotoxin Quantitation Kit (Thermo Scientific, UK). Peptide functionality was confirmed by assessing anti-endotoxic activity [14].

### Cell culture

HEp-2 cells (ATCC CCL-23), Vero cells (ATCC CCL-81) and 16HBE14o^-^ transformed human bronchial epithelial cells (a kind gift from Dieter Gruenert, University of California [15]) were cultured in DMEM/F12 (Gibco/Life Technologies Ltd, Paisley, UK) or RPMI (Gibco) respectively, supplemented with 2 mM L-glutamine (PAA Laboratories, Yeovil, UK) and 10% FCS (Biosera, East Sussex, UK), at 37°C and 5% CO_2_. 16HBE14o^-^ cells were cultured on wells previously coated with a basement layer of collagen IV (5 µg/ml), fibronectin (10 µg/ml) and bovine serum albumin (100 µg/ml) (all Sigma Aldrich, Dorset, UK).

### Virus propagation

RSV strain A2 (VR-1540) was purchased from ATCC. RSV was propagated in HEp-2 cells by infecting 5×10^6^ cells that were allowed to grow on confluent overnight in serum-free VP-SFM media (Gibco) for 2 hours with 0.1 plaque forming units (PFU) followed by removal of inoculum. Cells were then incubated for 24 hours in 10% FCS Gold (PAA Laboratories) supplemented VP-SFM. Cells were then incubated for a further 5 days in 2% FCS Gold supplemented VP-SFM, cells were harvested and supernatant was snap-frozen and stored at −80°C.

### Immunoplaque assay

An immunoplaque assay (based on [16]) was used to quantify RSV infected cells. Optimisation of the infection protocol was conducted using serial dilutions of RSV to assess the PFU of propagated virus stocks and establish an optimal MOI of 0.005 that allowed reliable counting of individual infected cells (approximately 100–250 per well) in a control infected culture at 24 hours. Peptide treatments were therefore primarily evaluated at RSV MOI = 0.005, however higher MOIs (up to 1) were also tested and similar effects of peptides were observed (data not shown). For experimental assessment of the antiviral effects of peptides, cells were seeded at 2×10^4^ per well in 96- well plates and cultured overnight, then washed and infected, in fresh serum-free medium. After 2 hours, inoculum was removed and cells were cultured in fresh medium for 24, 48 or 72 hours before analysis. Peptides were added before, after or concomitantly with RSV infection as described for individual experiments. Monolayers were fixed in absolute methanol/2% hydrogen peroxide solution for 30 minutes and washed twice with PBS. Cells were exposed to monoclonal biotinylated anti-RSV antibody in 5% BSA/PBS for 1 hour (1∶200, AbD Serotec, Kidlington, UK), washed twice with PBS and were incubated with extravidin peroxidase in 5% BSA/PBS for 30 minutes (1∶500, Sigma Aldrich). Cells were stained using 3-amino-ethylcarbazole substrate (Invitrogen/Life technologies) according to manufacturer's instructions for 15 minutes, washed 3 times with PBS and infected RSV positive foci were quantified by light microscopy, counting 3–4 wells per condition. For later timepoint analysis (48 and 72 hours post infection with RSV MOI = 0.0005), fixed and stained monolayers were examined by light microscopy and images were captured using an EVOS fl imaging system (PEQLAB Ltd, UK). Quantification was performed using the ImageJ plugin Treshold_colour to measure the percentage of cells positive for RSV infection. 3 wells were assessed per condition, using 4 random fields of view captured per well. To assess the production of infectious viral particles, supernatants were serially diluted and transferred to fresh HEp-2 monolayers, incubated for 2 hours at 37°C, washed, incubated for a further 24 hours and then assessed by immunoplaque assay.

### Direct viral inactivation assay

A modified viral inactivation assay [17] was performed. 1 ml RSV (PFU = 2×10^3^, equivalent to MOI = 0.05) was mixed with 25 µg/ml LL-37, followed by immediate 10-fold dilution in serum-free medium to reach sub-therapeutic concentrations of LL-37 (2.5 µg/ml) and RSV MOI = 0.005. 25 µg/ml scrambled LL-37 or PBS were used as negative controls. HEp-2 monolayers were infected with this virus/peptide mixture for 2 hours then washed, before cells were cultured in serum-free medium for 24 hours. Fixed monolayers were subjected to immunoplaque assay.

### Visualisation of TAMRA-labelled LL-37

HEp-2 cells were treated with unlabelled- or red-fluorescent tetramethylrhodamine (TAMRA)-labelled LL-37 at 25 µg/ml for 1 hour, followed by a PBS wash. Hoechst stain was added at 1 µg/ml for 30 minutes to visualise nuclei, and live cells were assessed by confocal microscopy, using a Leica SP5 confocal microscope with a 63× objective and Leica Application Suite Advanced Fluorescence software. A minimum of 4 fields of view were assessed for each condition.

### Western blotting

HEp-2 cells were treated with LL-37 for 1 hour, or left untreated, followed by PBS washes or left unwashed. Cells were lysed on ice using lysis buffer (20 mM Hepes, 0.3 mM NaCl, 1.5 mM MgCl2, 0.2 mM EDTA, 1 mM DTT, 1 mM Orthovanadate, 0.5% TritonX100) with Complete Protease Inhibitor Cocktail tablets (Roche Applied Science, UK; 1 tablet dissolved in 1 ml ddH2O and used at a 1 in 50 dilution). A BCA test was used to determine protein concentration, as per manufacturer's instructions (Thermo Scientific, UK) and equal amounts of protein were incubated (5 min, 95°C) with sample buffer and reducing agent (Invitrogen). SDS polyacrylamide gel electrophoresis was performed, using 16% denaturing gels (100 V, 5 min, then 120 V, 85 min), before proteins were transferred to PVDF membranes by electro-blotting (100 V, 90 min). Membranes were blocked with 5% milk in PBS for 1 hour and incubated with LL-37 primary antibody (Innovagen AB, Lund, Sweden; 1∶1000, 5% milk/PBS) overnight. Following 3 washes in Tris buffered saline with 0.05% Tween 20 (TBST), membranes were exposed to extravidin-peroxidase coupled secondary antibody (mouse anti-rabbit, Jackson ImmunoResearch, Suffolk, UK; 1∶10,000) for 1 hour, then washed with TBST. Chemiluminescence substrate (GE Healthcare Life Sciences, Buckinghamshire, UK) was added to membranes for 1 minute and the resulting chemiluminescence was detected by exposition to film.

### Assessment of cytopathic effects

HEp-2 cells were treated with LL-37, a scrambled control LL-37 at 50 µg/ml or ribavirin (200 µM; Sigma Aldrich), in the presence or absence of simultaneous infection with RSV (MOI = 0.005). Cells were incubated for 2 hours at 37°C, washed and placed in fresh media. In some wells LL-37 or ribavirin was added again at this point (for “continuous treatment”). Cells were cultured at 37°C for 24 hours or 5 days, after which the number of viable metabolically active cells was assessed using an MTT assay (Sigma Aldrich) according to manufacturer's instructions (7.5 mg/ml, 37°C, 4 hours). Resulting formazan was dissolved in dimethylsulfoxide (Sigma Aldrich) and optical density was measured at 450 nm. Alternatively, cells simultaneously treated with or without LL-37 in presence or absence of RSV infection were allowed to dry for 10 minutes before they were fixed and permeabilised using ice-cold acetone/methanol (1∶1) for 10 minutes at 4°C. After a wash with PBS, cells were subjected to TUNEL assay using an In situ cell death kit (Roche Applied Science, UK) according to manufacturer's instructions. Cells were washed three times with PBS and 1 µg/ml DAPI was included in the second wash (3 minutes staining). Cells were examined by fluorescent microscopy, four representative images per well were captured using an EVOS fl imaging system (PEQLAB Ltd) and TUNEL positive and negative cells were quantified using ImageJ (http://rsbweb.nih.gov/ij/index.html).

### Statistical analysis

Statistical analyses were performed as described using Graphpad Prism version 5 for Windows (GraphPad Software, Inc., La Jolla, CA, USA). For statistical analysis, immunoplaque counts were log transformed. Statistical significance was assessed either using a one-way analysis of variance (ANOVA) with Dunnett Multiple Comparison post test, or a two-way ANOVA with Bonferroni's post test where appropriate. p≤0.05 was considered significant.

## Results

### LL-37 has antiviral activity against RSV

In order to assess the antiviral potential of LL-37 against RSV, HEp-2 human epithelial cells were infected with RSV and concomitantly treated with a concentration range of LL-37 peptide, then assessed by counting the number of RSV- infected cells by immunoplaque assay after 24 hours. This simultaneous exposure to virus and LL-37 resulted in a significant, concentration-dependent reduction in infectivity at ≥10 µg/ml of LL-37 (p<0.05), compared to virus alone (Figure 1b). The same effect was observed when the human bronchial airway epithelial cell line 16HBE14o^-^ was used (Figure 1c), demonstrating that the effect was not specific to the HEp-2 cell line. A scrambled LL-37 control peptide had no antiviral effect (Figure 1c), demonstrating that this was not a non-specific effect of a cationic peptide. In order to determine whether maximal antiviral effect required interaction between the peptide and virus in advance of infection, the sequence of peptide and virus addition to cell was then modified. As a mimic of the physiological encounter between RSV and LL-37 that might be expected to occur in the airway surface liquid *in vivo* before reaching epithelial cells, virus was pre-incubated with LL-37 (at 37°C or at 4°C, given the temperature sensitive nature of RSV[18]), before HEp-2 cell infection. These conditions, in which peptide may have effects on the cells, the virus or both, also resulted in significant antiviral activity (Figure 1d; LL-37 effect p<0.05 by one-way ANOVA), resulting in approximately 1 log fewer infected cells at 25 µg/ml of LL-37 (p<0.01 at 37°C and p<0.001 at 4°C), but not substantially different to the simultaneous addition of peptide and virus without preincubation. In contrast, in these experiments, delaying LL-37 treatment until 2 hours after viral infection resulted in a loss of the antiviral effect (Figure 1d), indicating that LL-37 treatment after infection does not “rescue” infected cells. However, pre-incubation of HEp-2 cells with LL-37, followed by cell washing prior to infection, also resulted in significantly less RSV infection (p<0.001; Figure 1d). These data demonstrate that LL-37 has significant antiviral activity against RSV infection of epithelial cells *in vitro* and suggest that this peptide may exert its effects by directly affecting the viral particle and/or by acting on epithelial cells to reduce their susceptibility to infection.

### The antiviral effects of LL-37 are protective against cell death

**Figure 2:**
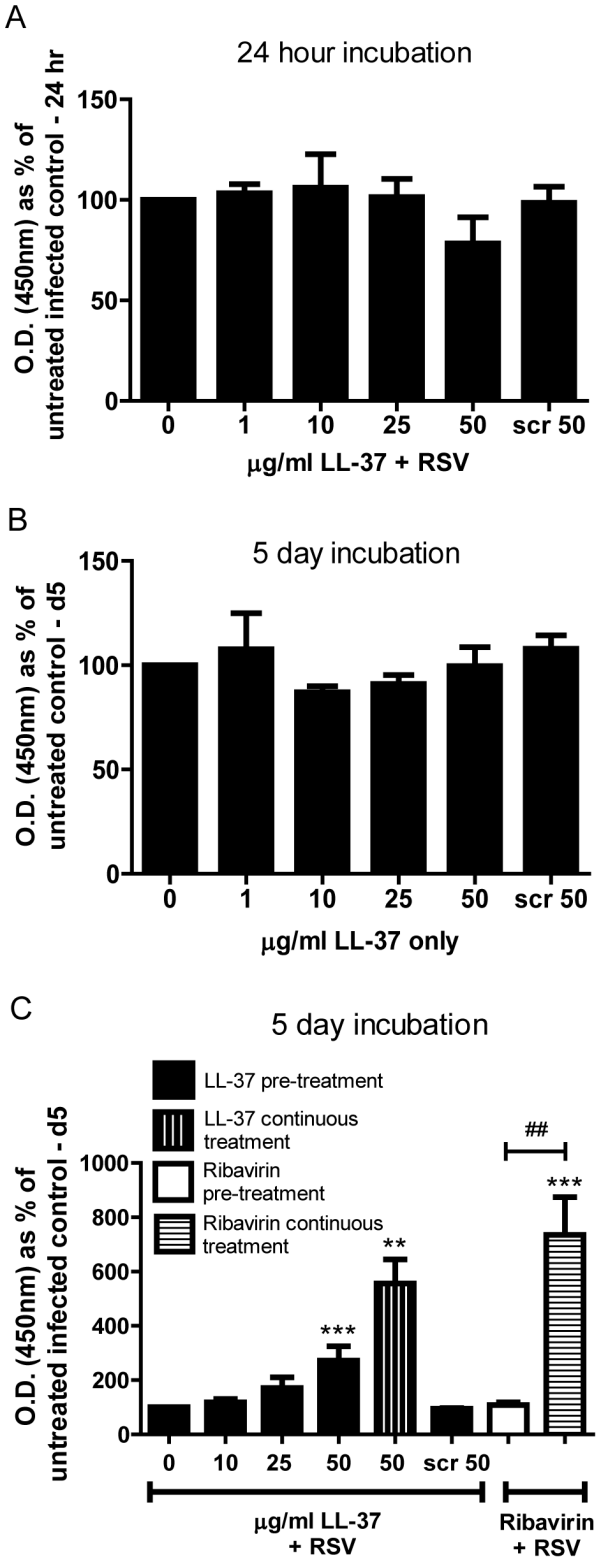
LL-37 is protective against RSV-induced cell death. HEp-2 cells were treated with LL-37 at 0–50 µg/ml, a scrambled control LL-37 at 50 µg/ml (Scr 50), or ribavirin (200 µM), in the presence (a & c) or absence (b) of simultaneous infection with RSV (MOI = 0.005). Cells were incubated for 2 hours at 37°C, washed and placed in fresh medium. In some wells LL-37 or ribavirin was added again at this point (c; “continuous treatment”). Cells were cultured at 37°C for 24 hours (a) or 5 days (b & c), after which the number of viable metabolically active cells was assessed using an MTT assay with measurement of optical density at 450 nm, expressed as a percentage of untreated infected (a & c) or untreated (b) cells. Results are shown as mean +/− SEM of n = 3-4. The data were analysed by 1-way ANOVAs with Dunnett Multiple Comparison Post-tests comparing peptide concentrations against the “no peptide” control (** p<0.01, *** p<0.001), and 2-way ANOVA with Bonferroni Post-tests comparing pre-treatment protocols versus continuous treatments (## p<0.01).

We have previously demonstrated that LL-37 can induce apoptosis in *Pseudomonas aeruginosa*-infected epithelial cells [19]. In order to determine whether similar mechanisms could lead to reduced infection through epithelial cell death, or whether LL-37 was actively protecting cells from infection, we examined the number of viable, metabolically active HEp-2 cells by MTT assay and induction of cell death by TUNEL assay. The treatment of RSV-infected cells with LL-37 (≤50 µg/ml) did not have any substantial effect on the number of metabolically active HEp-2 cells at 24 hours (Figure 2a), nor any substantial effects on the low background levels of apoptosis in this cell line (0.5–1.5% of HEp-2 cells were found to be TUNEL positive in the presence or absence of RSV or LL-37 at 24 hours after infection; data not shown). In order to determine longer term effects, treated cells were examined for up to 5 days. The treatment of uninfected cells with LL-37 (≤50 µg/ml) did not affect the number of metabolically active HEp-2 cells, even after 5 days (Figure 2b). In contrast LL-37 was found to be protective in RSV infected cells (Figure 2c). HEp-2 cells were simultaneously incubated with RSV and LL-37 or RSV and the antiviral agent ribavirin (as a positive control) for two hours, before being washed and incubated for five days in the absence of LL-37/ribavirin (Figure 2c; “pre-treatment” condition). Notably, LL-37 treatment resulted in significantly more viable HEp-2 cells surviving RSV infection in a dose-dependent manner (overall LL-37 effect p<0.001 by 1-way ANOVA). Neither scrambled LL-37 nor the antiviral ribavirin had any direct effect under these conditions. Since ribavirin prevents viral replication, rather than cell infection, the effect of continuous presence of treatment in the media over the five day study was evaluated (Figure 2c; “continuous treatment” condition). Continuous treatment of RSV-infected HEp-2 cells with either ribavirin or LL-37 resulted in a significant protection of epithelial cells from cell death compared to the untreated infected cells (LL-37 p<0.01 and ribavirin p<0.001), with substantially greater protective effects than observed in the pre-treatment condition (significantly different for ribavirin; p<0.01, but not reaching statistical significance for LL-37). These data demonstrate that LL-37 can protect against RSV-induced cytopathic effects.

### LL-37 directly affects RSV viral particles

**Figure 3:**
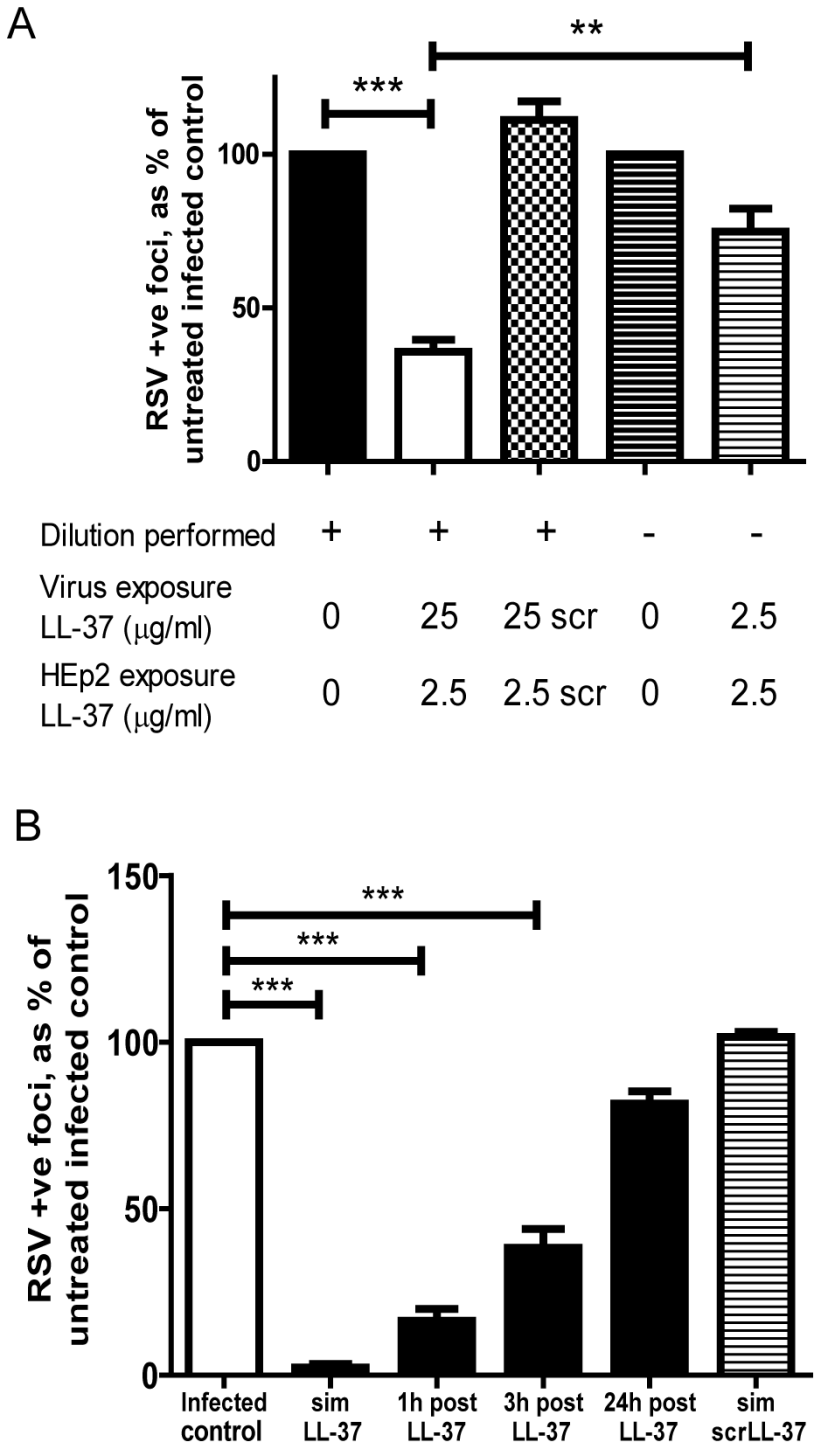
LL-37 has protective antiviral effects by acting both on viral particles and cells. a) RSV (MOI = 0.05) was pre-mixed with LL-37, or a scrambled LL-37 control peptide (scrLL-37) at 25 µg/ml, followed immediately by a 1∶10 dilution that was used to infect HEp-2 cells for 2 hours or RSV (MOI = 0.005) was pre-mixed with LL-37 at 2.5 µg/ml before immediate use. b) HEp-2 cells were simultaneously treated for 2 hours with RSV (MOI = 0.005) and LL-37 (“sim LL-37” condition), or a scrambled LL-37 control peptide (“sim scrLL-37” condition), at 25 µg/ml, or were pre-treated with 25 µg/ml LL-37 for 1 hour, washed and then infected with RSV (MOI = 0.005, 2 hours) at 1, 3 or 24 hours after initial exposure to LL-37. Cells were washed and cultured for 24 hours at 37°C, then the number of infected cells was quantified using an immunoplaque assay and expressed as a percentage relative to untreated, RSV infected cells. Results are shown as mean +/− SEM of n≥3. For statistical analysis, RSV-positive foci counts were log transformed and analysed by a) two-way ANOVA with Bonferroni post-test to compare LL-37 treatment with appropriate control and the effects of dilution, or b) one way ANOVA with Dunnett Multiple Comparison Post-tests to compare treatments to untreated infected controls; ** p<0.01, *** p<0.001.

In order to determine whether LL-37 could have a direct effect upon RSV viral particles, a concentrated viral stock was exposed to LL-37 at 25 µg/ml, immediately followed by a 1 in 10 dilution, reducing the concentration of LL-37 to which cells were exposed to below the effective antiviral level (to 2.5 µg/ml) before incubation with HEp-2 cells. This was compared to pre-mixing of virus (at standard concentration) with LL-37 at 2.5 µg/ml before infection of HEp-2 cells (Figure 3a). The combination of virus exposure to 25 µg/ml LL-37, with cell exposure to 2.5 µg/ml LL-37, had significant antiviral effects (p<0.001), whereas the combination of virus and cell exposure to 2.5 µg/ml LL-37 had no significant effects and was significantly different from the former (p<0.01). No effect was observed using scrambled LL-37 under the same dilution conditions. These data demonstrate that the antiviral properties of LL-37 against RSV are at least partly due to a direct interaction with the viral particles, an effect that was separable, under these conditions, from any peptide-mediated effects on the cells.

### LL-37 modulates epithelial cell susceptibility to RSV infection

Having demonstrated that pre-treatment of epithelial cells with LL-37 could protect against subsequent infection by RSV (Fig. 1c), the longevity of that protective effect was determined by delaying RSV infection of cells for a variable period of time after LL-37 treatment and washing of cells (Figure 3b). A significant protection against RSV infection was observed for up to 3 hours after LL-37 treatment and cell washing (p<0.001). However, a time dependent loss of protection was observed, with no protection evident 24 hours after LL-37 treatment and cell washing.

**Figure 4:**
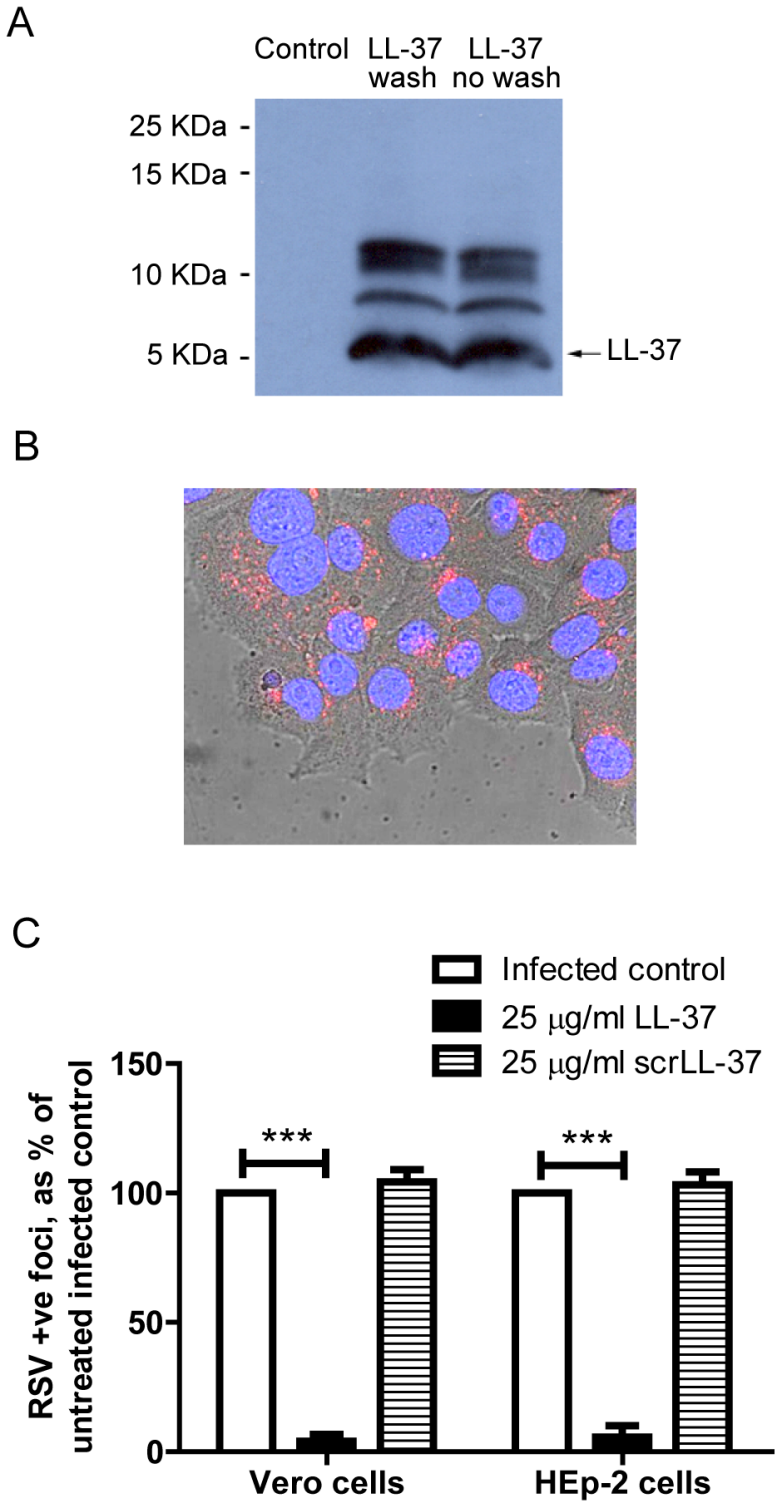
LL-37 is retained by epithelial cells and does not act via modulation of type I interferon production. HEp-2 cells were treated with 25 µg/ml of LL-37 (a) or TAMRA-labelled LL-37 (b) for 1 hour, or left untreated. a) LL-37 treated cells were then subjected to repeated wash steps or left unwashed, before cell lysis and protein collection. The presence of LL-37 was assessed by western immunoblot. Data is representative of n = 3 independent experiments. b) TAMRA-labelled LL-37 treated cells were washed and then Hoechst staining was added before imaging by confocal microscopy. A merged brightfield/Hoechst/TAMRA-LL-37 image is shown, nuclei in blue and TAMRA-labelled LL-37 in red. Image is at 63× magnification. c) Vero cells and HEp-2 cells were infected with RSV (MOI = 0.005) and simultaneously treated with LL-37 or a scrambled control LL-37 (scrLL-37) at 25 µg/ml for 2 hours. Cells were washed, incubated for 24 hours at 37°C, then the number of infected cells was quantified using an immunoplaque assay and expressed as a percentage relative to untreated, RSV infected cells. Results are shown as mean +/− SEM of n = 3-5. For statistical analysis, RSV-positive foci counts were log transformed and analysed by one way ANOVA with Dunnett Multiple Comparison Post-tests to compare treatments to untreated infected controls *** p<0.001.

The pre-treatment of cells with LL-37, followed by washing of the cells, was conducted in an effort to remove LL-37 from the cell culture system before addition of RSV. However, we have previously demonstrated that LL-37 can interact with epithelial cells and be actively transported into the cell [20]. Thus, whole cell lysates were examined by western immunoblot for LL-37 before and after cell washing (Figure 4a). No expression of hCAP18 (18 kDa) was observed in untreated cells or treated cells. No difference in the levels of exogenous LL-37 were observed between washed and unwashed peptide treated cells, indicating that washing did not remove the peptide from the HEp-2 cells. In order to confirm retention of the peptide by HEp-2 cells, cells were exposed to a TAMRA-labelled LL-37, washed and then imaged by fluorescent confocal microscopy (Figure 4b). TAMRA-labelled LL-37 was clearly observed to be associated with the epithelial cells, but not with the tissue culture plastic. Confocal z-stack analysis indicated that this peptide was predominantly intracellular (data not shown) with perinuclear distribution (Figure 4b).

The activity of the internalised peptide is unclear, but an intracellular receptor (GAPDH) for LL-37 has been proposed [21], and the antiviral effect of LL-37 pre-treatment of cells raised the possibility that LL-37 might induce a protective antiviral state in treated epithelial cells. As LL-37 has been shown to modulate expression of antiviral type I interferons in other systems [22] experiments were repeated in the type I interferon deficient [23] Vero cell line (Figure 4c). LL-37 demonstrated identical significant antiviral activity (p<0.001) against RSV in both cell lines, indicating that modulation of type I interferons was not required for the protective effect of LL-37. These data demonstrate that cell-associated LL-37 can mediate protection from RSV infection, likely independently of the direct activity on viral particles, but the mechanism by which this cell-associated peptide protects the epithelial cells remains to be determined.

### LL-37 can protect epithelial cells against spread of virus infection

**Figure 5:**
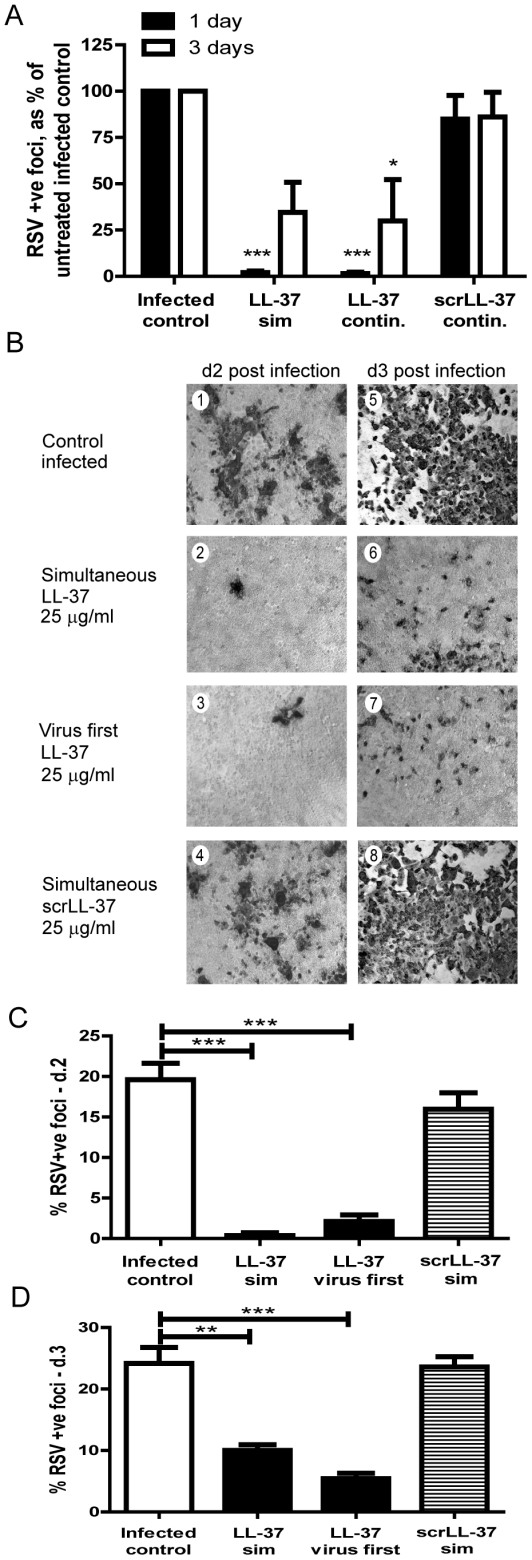
LL-37 inhibits the production of new viral particles and the spread of infection. a) HEp-2 cells were simultaneously exposed to RSV with either 25 µg/ml LL-37 or scrambled LL-37 (scrLL-37) for 2 hours, washed and incubated in fresh media with (“continuous”), or without (“sim”) replacement of LL-37 or scrambled LL-37 at 25 µg/ml, at 37°C for 1 day or 3 days. Supernatants were then transferred in serial dilution to infect fresh HEp-2 cells and incubated for 2 hours, before these cells were incubated for 24 hours and assessed by immunoplaque assay, expressed as a percentage relative to those infected with supernatant from untreated, RSV infected cells. Results are shown as mean +/− SEM of n = 3. For statistical analysis, RSV-positive foci counts were log transformed and analysed by one way ANOVA with Dunnett Multiple Comparison Post-tests to compare treatments to untreated infected controls * p<0.05, *** p<0.001, b, c & d) HEp-2 cells were either infected with RSV alone (MOI = 0.0005) for 2 hours (b, panels 1 & 5), simultaneously exposed to RSV with either 25 µg/ml LL-37 (b, panels 2 & 6; c & d, “LL-37 sim”) or scrambled LL-37 (b, panels 4 & 8; c & d, “scrLL-37 sim”) for 2 hours, or incubated with RSV alone for 2 hours, then washed, before 25 µg/ml LL-37 was added for 1 hour (b, panels 3 & 7; c & d, “LL-37 virus first”). Subsequently, cells were washed and cultured in fresh media for 48 hours (b, panels 1–4; c) or 72 hours (b, panels 5–8; d) at 37°C, before immunoplaque assay. Extent of infection in different treatments was evaluated by light microscopy with infected cells staining dark (b). Images are representative of n = 3 independent experiments. Magnification ×40. c & d) Areas of infection on light micrographs were quantified from 4 random fields per sample from each of n = 3 independent experiments using Image J. Data represent +/− SEM of n = 3. Analysis was performed by one way ANOVA with Dunnett Multiple Comparison Post-tests to compare treatments to untreated infected controls ** p<0.01, *** p<0.001.

In order to examine the effects of LL-37 on the production of new infectious viral particles, HEp-2 cells were infected with RSV in the presence or absence of LL-37 for 2 hours, then cultured in fresh media for up to 3 days, with or without the continued presence of LL-37 or scrambled LL-37. At selected timepoints, infectivity of the supernatant from these cells was assessed for its ability to infect fresh HEp-2 cell cultures (Figure 5a). Treatment with LL-37 (but not scrambled LL-37) resulted in the production of significantly (p<0.001), and very substantially, fewer new infectious viral particles, even reducing infectivity up to day 3 (statistically significantly when LL-37 remained in the media; p<0.05).

In the previous experiment (Figure 1d), addition of LL-37 at 2 hours after RSV infection had no effect on the number of infected cells at 24 hours. However, the effect on production of new infectious viral particles suggested that LL-37 might prevent the spread of infection from initially infected cells to uninfected cells in the culture over a longer time course. In order to examine this *in vitro*, HEp-2 cells were infected with a lower inoculum and evaluated by immunoplaque assays over 3 days (Figure 5b). The number of infected cells dramatically increased by day 2 in untreated cultures (Figure 5b, panel 1) and those exposed to scrambled LL-37 (Figure 5b, panel 4), rapidly reached uncountable levels of infected cells with death and substantial loss of cells by day 3 (Figure 5b, panels 5 & 8). In contrast, this spread of infection beyond the cells initially infected was minimised when LL-37 had been added simultaneously with RSV (Figure 5b, panels 2 & 6), or indeed even after RSV infection had been established (Figure 5b, panels 3 & 7). A quantitative image analysis approach to these data significantly underestimates the number of infected cells where cell death and loss has occurred, but nevertheless demonstrates significant protection against spread of infection from initially infected cells over 2 to 3 days, regardless of whether LL-37 was added concomitantly with, or after viral infection (Figures 5c & d). These data demonstrate that LL-37 can significantly inhibit the spread of newly produced infectious particles.

### The antiviral activity of LL-37 is retained by a truncated peptide fragment

**Figure 6:**
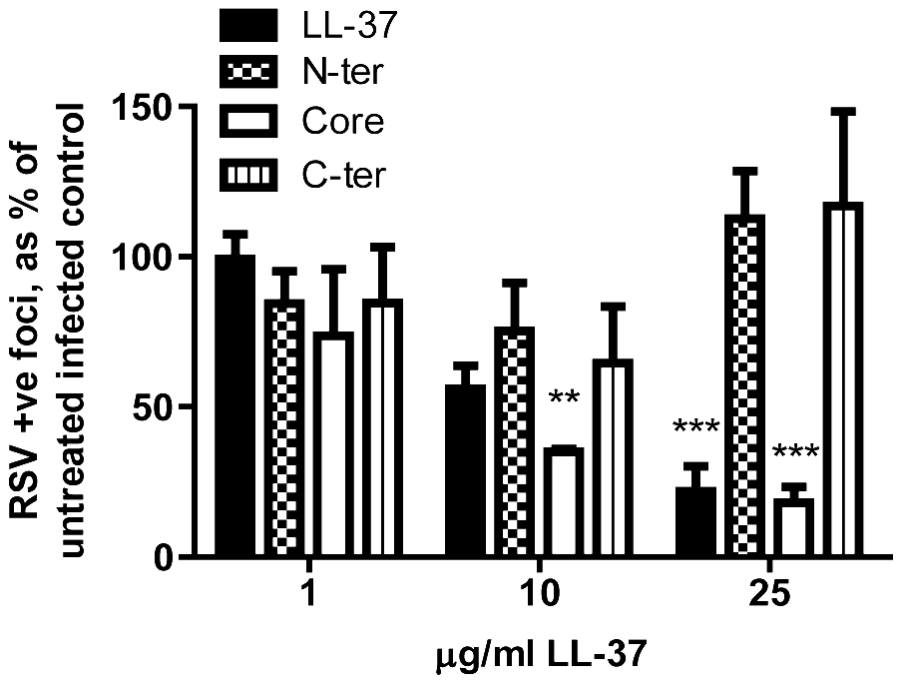
The antiviral activity of LL-37 is retained by a central truncated fragment. HEp-2 cells were simultaneously treated with RSV (MOI = 0.005) and full length LL-37 or one of three 22-mer peptide fragments (all at 1 µg/ml, 10 µg/ml, and 25 µg/ml; see Figure 1a) for 2 hours before washing and 24 hours culture at 37°C. The number of infected cells was quantified using an immunoplaque assay and expressed as a percentage relative to untreated, infected cells. Results are shown as mean +/− SEM of n = 3. For statistical analysis, RSV-positive foci counts were log transformed and compared to no peptide treatment, analysed by 2-way ANOVA with Bonferroni Post-tests for specific peptide concentrations ** p<0.01, *** p<0.001.

Having established the anti-RSV activity of LL-37, partial LL-37 peptides were utilised to determine the antiviral properties of truncated forms. Three 22-mer peptides [24] were used, which correspond to the N-terminal (amino acids 1–22), central core (amino acids 12–33) and C-terminal (amino acids 16–37) sequence of the LL-37 peptide (Figure 1a). Simultaneous treatment of HEp-2 cells with RSV and peptides from this panel revealed significant antiviral activity for full length LL-37 and the central core fragment, but no effects for the N-terminal and C-terminal fragments (Figure 6). These data demonstrate that shorter peptide analogues, more amenable to the development of novel therapeutics, retain antiviral activity.

## Discussion

RSV is a major cause of morbidity and mortality globally, particularly in infants, for which there is currently no effective vaccination or treatment available. In experimentally induced RSV infection in adult volunteers, early high viral titres have been found to correlate with severity of inflammatory disease manifestations [25]. In addition, more severe disease in infants is associated with high viral titres in early stages of naturally-occurring RSV bronchiolitis [26]. Thus, components of innate host defence with the capacity to reduce viral load would be expected to prevent or reduce severe disease. Here, we demonstrate for the first time that the cationic host defence peptide LL-37 has effective antiviral activity against RSV *in vitro*, implicating this peptide as a targetable host defence factor with a potentially key role in susceptibility to RSV infection *in vivo*.

LL-37 is a pleiotropic CHDP, cleaved from the sole human cathelicidin hCAP-18. This peptide can be detected in a broad range of tissues and bodily fluids including plasma, bone marrow, airway surface fluid, skin, sweat, reproductive tract, semen, urine, breast milk and vernix (reviewed in [27]). Produced primarily by neutrophils and epithelial cells, hCAP-18/LL-37 is upregulated in response to infection, inflammation and wounding, and detected at higher concentrations in the bronchoalveolar lavage fluid of individuals in the context of lung infections, reaching average levels of approximately 20–25 µg/ml [28], [29]. More specifically, the expression of *CAMP* (the gene encoding hCAP-18) can be regulated by the active vitamin D metabolite 1,25-dihydroxyvitamin D3 (1,25OH Vit D3), acting via a vitamin D response element in the *CAMP* gene promoter [12], [30], [31], an effect that can promote RSV-induced hCAP-18 production [11]. In addition to well described bactericidal properties, we, and others, have recently demonstrated that LL-37 has antiviral activity against a number of viruses (reviewed in [7]), including influenza *in vitro* [32], [33] and *in vivo* [32]. Although the specific mechanisms involved remain largely undetermined.

We now demonstrate that LL-37 has concentration-dependent antiviral activity against RSV *in vitro*, capable of decreasing the number of initially infected cells by approximately 1 log at the physiologically relevant [28], [29] concentration of 25 µg/ml. LL-37 prevented virus-induced cell death in epithelial cultures, significantly inhibited the production of new infectious particles and diminished the spread of infection. The effect was maximal when peptides were premixed with virus, or added simultaneously. However, significant depression of the extent of viral infection after three days was seen (Figure 5) even when the peptide was not added until two hours after infection of the cells, despite no effect on the number of entry events being observed at 24 hours under these conditions (Figure 1c). The specific events in the infection cycle that are affected by LL-37 remain to be determined. Nevertheless, these data suggest that higher baseline endogenous levels of LL-37 and/or enhanced production of LL-37 in response to RSV infection would offer protection in the lung. This hypothesis was validated by the recent study demonstrating clinical associations between low serum cathelicidin levels in children and both the likelihood of RSV bronchiolitis and the severity of disease [13]. These studies raise the possibility that interventions capable of enhancing upregulation of LL-37 levels *in vivo* could be used in RSV infection, given prophylactically in high risk individuals, such as infants predisposed to repeated RSV bronchiolitis.

The development of strategies to induce LL-37 expression is currently focused on the use of drugs such as butyrate and/or the use of vitamin D supplementation (reviewed in [34]). Butyrate is a short-chain fatty acid produced by bacterial fermentation of dietary fibre in the colon. 4-phenylbutyrate (PBA), an analogue of butyrate that is already licenced for use in humans, can effectively upregulate hCAP-18/LL-37 expression *in vitro*, including in airway epithelial cells [35], and *in vivo* in a model of Shigella infection [36] and has potential where more acute, short term upregulation is required. Low serum 25(OH) VitD3 levels, and the decrease in levels observed during winter [37], are associated with the risk of respiratory infections, with a 7% reduction in adult respiratory infection reported for every 10 nM/l increase in serum 25(OH) VitD3 reported [38]. In addition, newborns with low cord blood 25(OH) VitD3 levels were found to have a significantly greater risk of developing RSV-associated lower respiratory tract infections [39]. Expression of hCAP-18/LL-37 can be regulated by vitamin D [12], [30], [31] and further upregulated by exposure to RSV in the presence of vitamin D [11]. An association between serum 25(OH) VitD3 and hCAP-18/LL-37 expression has not yet been definitely demonstrated *in vivo*. Nevertheless, these studies raise the possibility that vitamin D supplementation (particularly in winter in the northern hemisphere), should be considered as a novel preventative strategy against RSV infection. Furthermore, LL-37 expression may represent a candidate mechanism involved in understanding these observations.

In addition to prophylactic modulation of LL-37 expression, our data raise the possibility that LL-37 peptide administration may also have potential in post-exposure prophylaxis, given in the early stages after infection to minimise the spread of RSV in the epithelium, viral load and thus the severity of disease. *In vivo* modelling is required to evaluate the efficacy of such an approach, but the observation that antiviral activity is retained by a truncated peptide demonstrates the potential to develop shorter synthetic analogues for future testing. Interestingly, our evaluation of these previously characterised [24] partial peptides representing the N-terminus (amino acids 1–22), a central portion (amino acids 12–33) and the C-terminus (amino acids 16–37) of LL-37 suggests that the antiviral activity may relate to the same core peptide region previously identified for antibacterial activity [24], [40] and for the capacity to induce secondary necrosis of apoptotic cells [41].

The specific mechanisms by which LL-37 has antiviral activity against RSV are now under evaluation, but remain to be determined. Our data demonstrate that LL-37 is capable of having a direct effect on the viral particles. This was recently demonstrated to occur in LL-37 treatment of influenza virus [33]. However, damage to the influenza viral membrane did not alter the binding or initial uptake of virus by cells, but was proposed to affect viral propagation or survival downstream of this. Interestingly, similar to our observations with RSV, LL-37 was also found to have antiviral activities against influenza when cells where exposed to peptide in advance of infection [33]. However, in contrast, LL-37-dependent inhibition of HIV-1 infectivity is mediated by inhibition of HIV-1 reverse transcriptase and protease and does not require direct peptide-virus interaction [42], [43]. Furthermore, LL-37-mediated antiviral activity against the non-enveloped adenovirus has also been reported [44], suggesting important activities other than direct damage to viral envelopes.

Our data demonstrate that LL-37 treatment in advance of viral infection results in peptide retention and internalisation by the epithelial cells, providing significant protection for at least 3 hours after cell washing. This is in keeping with our previous observations of active cellular uptake of LL-37 [20], and suggests that either RSV may interact with cell-associated LL-37 or that LL-37 may induce an antiviral state in the epithelial cells. Interestingly, human β-defensin (hBD)2 has been shown to destabilise the RSV viral envelope upon contact with soluble hBD2 in solution, or following exposure to plasma membrane-associated hBD2 during cell entry [45]. Although LL-37 may function in a similar manner, the well described capacity of LL-37 to modify cellular responses to inflammatory stimuli raises the possibility that host defence modulatory capacity is important.

LL-37 has been demonstrated to modulate signalling downstream of pattern recognition receptors and differentially induce and inhibit certain cytokine and chemokines responses, with the potential to modify the nature of host responses to bacterial and viral infections [14], [46], [47]. Interestingly these effects are observed at peptide concentrations (typically 1–5 µg/ml) that are lower than those required for microbicidal activity. Although our data indicate that LL-37-mediated modulation of cellular type –I interferon responses is not required for the antiviral activity against RSV observed in this study, the capacity of cathelicidins to modulate cellular responses to TLR3, −7, −8 and −9 agonists [22], [47], [48] may prove to be significant. Indeed, the dramatic effects of LL-37 on cytokine production and survival in influenza infected mice, in comparison to the relatively modest effect on viral loads *in vivo* [32], also suggests that peptide-mediated modulation of inflammation and immunity may be an important component of their antiviral function. In addition to evaluating the impact of cathelicidins *in vivo* and the capacity for direct and indirect antiviral activities of these peptides to act simultaneously, future studies should also determine the extent to which synergies exist between cathelicidins and other CHDP (such as defensins) which have been shown to have activity against RSV.

In conclusion, this study demonstrates a novel antiviral activity for the sole human cathelicidin LL-37 against RSV, an important human pathogen for which no effective disease-modifying therapies exist. It implicates hCAP-18/LL-37 as an important, targetable component of innate host defence against RSV and suggests future potential in strategies aimed at prophylactic modulation of cathelicidin expression in vulnerable individuals and/or the development of synthetic peptide analogues for use in post-exposure prophylaxis.

## References

[1] Nair H, Nokes DJ, Gessner BD, Dherani M, Madhi SA, et al (2010) Global burden of acute lower respiratory infections due to respiratory syncytial virus in young children: a systematic review and meta-analysis. Lancet 375: 1545–1555. 20399493 10.1016/S0140-6736(10)60206-1 PMC2864404

[2] Falsey AR, Hennessey PA, Formica MA, Cox C, Walsh EE (2005) Respiratory syncytial virus infection in elderly and high-risk adults. N Engl J Med 352: 1749–1759. 15858184 10.1056/NEJMoa043951

[3] Smyth RL, Openshaw PJ (2006) Bronchiolitis. Lancet 368: 312–322. 16860701 10.1016/S0140-6736(06)69077-6

[4] Krishnamoorthy N, Khare A, Oriss TB, Raundhal M, Morse C, et al (2012) Early infection with respiratory syncytial virus impairs regulatory T cell function and increases susceptibility to allergic asthma. Nat Med 18: 1525–1530. 22961107 10.1038/nm.2896 PMC3641779

[5] Tregoning JS, Schwarze J (2010) Respiratory viral infections in infants: causes, clinical symptoms, virology, and immunology. Clin Microbiol Rev 23: 74–98. 20065326 10.1128/CMR.00032-09 PMC2806659

[6] Beaumont PE, Li H, Davidson DJ (2013) LL-37: An Immunomodulatory Antimicrobial Host Defence Peptide. In: Hiemstra PS, Zaat SAJ, Antimicrobial peptides and Innate Immunity. Basel: Springer. 97–122.

[7] Gwyer Findlay E, Currie SM, Davidson DJ (2013) Cationic Host Defence Peptides: Potential as Antiviral Therapeutics. BioDrugs May 7 . [Epub ahead of print]. 10.1007/s40259-013-0039-0 PMC3775153 23649937

[8] Lai Y, Gallo RL (2009) AMPed up immunity: how antimicrobial peptides have multiple roles in immune defense. Trends Immunol 30: 131–141. 19217824 10.1016/j.it.2008.12.003 PMC2765035

[9] Bowdish DME, Davidson DJ, Hancock REW (2006) Immunomodulatory properties of defensins and cathelicidins. Curr Top Microbiol Immunol 306: 27–66. 16909917 10.1007/3-540-29916-5_2 PMC7121507

[10] Sorensen OE, Follin P, Johnsen AH, Calafat J, Tjabringa GS, et al (2001) Human cathelicidin, hCAP-18, is processed to the antimicrobial peptide LL-37 by extracellular cleavage with proteinase 3. Blood 97: 3951–3959. 11389039 10.1182/blood.v97.12.3951

[11] Hansdottir S, Monick MM, Hinde SL, Lovan N, Look DC, et al (2008) Respiratory epithelial cells convert inactive vitamin D to its active form: potential effects on host defense. J Immunol 181: 7090–7099. 18981129 10.4049/jimmunol.181.10.7090 PMC2596683

[12] Yim S, Dhawan P, Ragunath C, Christakos S, Diamond G (2007) Induction of cathelicidin in normal and CF bronchial epithelial cells by 1,25-dihydroxyvitamin D(3). J Cyst Fibros. 10.1016/j.jcf.2007.03.003 PMC2099696 17467345

[13] Mansbach JM, Piedra PA, Borregaard N, Martineau AR, Neuman MI, et al (2012) Serum cathelicidin level is associated with viral etiology and severity of bronchiolitis. J Allergy Clin Immunol 130: 1007–1008.e1001. 22944482 10.1016/j.jaci.2012.07.044 PMC3462235

[14] Scott MG, Davidson DJ, Gold MR, Bowdish DME, Hancock REW (2002) The Human Antimicrobial Peptide LL-37 Is a Multifunctional Modulator of Innate Immune Responses. J Immunol 169: 3883–3891. 12244186 10.4049/jimmunol.169.7.3883

[15] Cozens AL, Yezzi MJ, Kunzelmann K, Ohrui T, Chin L, et al (1994) CFTR expression and chloride secretion in polarized immortal human bronchial epithelial cells. Am J Respir Cell Mol Biol 10: 38–47. 7507342 10.1165/ajrcmb.10.1.7507342

[16] Cannon MJ (1987) Microplaque immunoperoxidase detection of infectious respiratory syncytial virus in the lungs of infected mice. J Virol Methods 16: 293–301. 3312262 10.1016/0166-0934(87)90014-0

[17] Lin LT, Chen TY, Chung CY, Noyce RS, Grindley TB, et al (2011) Hydrolyzable tannins (chebulagic acid and punicalagin) target viral glycoprotein-glycosaminoglycan interactions to inhibit herpes simplex virus 1 entry and cell-to-cell spread. J Virol 85: 4386–4398. 21307190 10.1128/JVI.01492-10 PMC3126266

[18] Hambling MH (1964) Survival of the Respiratory Syncytial Virus during storage under various conditions. Br J Exp Pathol 45: 647–655. 14245166 PMC2093680

[19] Barlow PG, Beaumont PE, Cosseau C, Mackellar A, Wilkinson TS, et al (2010) The Human Cathelicidin LL-37 Preferentially Promotes Apoptosis of Infected Airway Epithelium. Am J Respir Cell Mol Biol 43: 692–702. 20097832 10.1165/rcmb.2009-0250OC PMC2993089

[20] Lau YE, Rozek A, Scott MG, Goosney DL, Davidson DJ, et al (2005) Interaction and cellular localization of the human host defense peptide LL-37 with lung epithelial cells. Infect Immun 73: 583–591. 15618198 10.1128/IAI.73.1.583-591.2005 PMC538997

[21] Mookherjee N, Lippert DN, Hamill P, Falsafi R, Nijnik A, et al (2009) Intracellular receptor for human host defense peptide LL-37 in monocytes. J Immunol 183: 2688–2696. 19605696 10.4049/jimmunol.0802586

[22] Lande R, Gregorio J, Facchinetti V, Chatterjee B, Wang YH, et al (2007) Plasmacytoid dendritic cells sense self-DNA coupled with antimicrobial peptide. Nature 449: 564–569. 17873860 10.1038/nature06116

[23] Desmyter J, Melnick JL, Rawls WE (1968) Defectiveness of interferon production and of rubella virus interference in a line of African green monkey kidney cells (Vero). J Virol 2: 955–961. 4302013 10.1128/jvi.2.10.955-961.1968 PMC375423

[24] Nell MJ, Tjabringa GS, Wafelman AR, Verrijk R, Hiemstra PS, et al (2006) Development of novel LL-37 derived antimicrobial peptides with LPS and LTA neutralizing and antimicrobial activities for therapeutic application. Peptides 27: 649–660. 16274847 10.1016/j.peptides.2005.09.016

[25] DeVincenzo JP, Wilkinson T, Vaishnaw A, Cehelsky J, Meyers R, et al (2010) Viral load drives disease in humans experimentally infected with respiratory syncytial virus. Am J Respir Crit Care Med 182: 1305–1314. 20622030 10.1164/rccm.201002-0221OC PMC3001267

[26] Scagnolari C, Midulla F, Selvaggi C, Monteleone K, Bonci E, et al (2012) Evaluation of viral load in infants hospitalized with bronchiolitis caused by respiratory syncytial virus. Med Microbiol Immunol 201: 311–317. 22406873 10.1007/s00430-012-0233-6 PMC7086883

[27] Bowdish DME, Davidson DJ, Hancock REW (2005) A re-evaluation of the role of host defence peptides in mammalian immunity. Curr Protein Pept Sci 6: 35–51. 15638767 10.2174/1389203053027494

[28] Schaller-Bals S, Schulze A, Bals R (2002) Increased Levels of Antimicrobial Peptides in Tracheal Aspirates of Newborn Infants during Infection. Am J Respir Crit Care Med 165: 992–995. 11934727 10.1164/ajrccm.165.7.200110-020

[29] Chen CI, Schaller-Bals S, Paul KP, Wahn U, Bals R (2004) Beta-defensins and LL-37 in bronchoalveolar lavage fluid of patients with cystic fibrosis. J Cyst Fibros 3: 45–50. 15463886 10.1016/j.jcf.2003.12.008

[30] Wang TT, Nestel FP, Bourdeau V, Nagai Y, Wang Q, et al (2004) Cutting edge: 1,25-dihydroxyvitamin D3 is a direct inducer of antimicrobial peptide gene expression. J Immunol 173: 2909–2912. 15322146 10.4049/jimmunol.173.5.2909

[31] Gombart AF, Borregaard N, Koeffler HP (2005) Human cathelicidin antimicrobial peptide (CAMP) gene is a direct target of the vitamin D receptor and is strongly up-regulated in myeloid cells by 1,25-dihydroxyvitamin D3. Faseb J 19: 1067–1077. 15985530 10.1096/fj.04-3284com

[32] Barlow PG, Svoboda P, Mackellar A, Nash AA, York IA, et al (2011) Antiviral Activity and Increased Host Defense against Influenza Infection Elicited by the Human Cathelicidin LL-37. PLoS One 6: e25333. 22031815 10.1371/journal.pone.0025333 PMC3198734

[33] Tripathi S, Tecle T, Verma A, Crouch E, White M, et al (2013) The human cathelicidin LL-37 inhibits influenza A viruses through a mechanism distinct from that of surfactant protein D or defensins. J Gen Virol 94: 40–49. 23052388 10.1099/vir.0.045013-0 PMC3542722

[34] van der Does AM, Bergman P, Agerberth B, Lindbom L (2012) Induction of the human cathelicidin LL-37 as a novel treatment against bacterial infections. J Leukoc Biol 92: 735–742. 22701042 10.1189/jlb.0412178

[35] Steinmann J, Halldorsson S, Agerberth B, Gudmundsson GH (2009) Phenylbutyrate induces antimicrobial peptide expression. Antimicrob Agents Chemother 53: 5127–5133. 19770273 10.1128/AAC.00818-09 PMC2786349

[36] Sarker P, Ahmed S, Tiash S, Rekha RS, Stromberg R, et al (2011) Phenylbutyrate counteracts Shigella mediated downregulation of cathelicidin in rabbit lung and intestinal epithelia: a potential therapeutic strategy. PLoS One 6: e20637. 21673991 10.1371/journal.pone.0020637 PMC3108617

[37] Sabetta JR, DePetrillo P, Cipriani RJ, Smardin J, Burns LA, et al (2010) Serum 25-hydroxyvitamin d and the incidence of acute viral respiratory tract infections in healthy adults. PLoS One 5: e11088. 20559424 10.1371/journal.pone.0011088 PMC2885414

[38] Berry DJ, Hesketh K, Power C, Hypponen E (2011) Vitamin D status has a linear association with seasonal infections and lung function in British adults. Br J Nutr 106: 1433–1440. 21736791 10.1017/S0007114511001991

[39] Belderbos ME, Houben ML, Wilbrink B, Lentjes E, Bloemen EM, et al (2011) Cord blood vitamin D deficiency is associated with respiratory syncytial virus bronchiolitis. Pediatrics 127: e1513–1520. 21555499 10.1542/peds.2010-3054

[40] Li X, Li Y, Han H, Miller DW, Wang G (2006) Solution Structures of Human LL-37 Fragments and NMR-Based Identification of a Minimal Membrane-Targeting Antimicrobial and Anticancer Region. J Am Chem Soc 128: 5776–5785. 16637646 10.1021/ja0584875

[41] Li HN, Barlow PG, Bylund J, Mackellar A, Bjorstad A, et al (2009) Secondary necrosis of apoptotic neutrophils induced by the human cathelicidin LL-37 is not proinflammatory to phagocytosing macrophages. J Leukoc Biol 86: 891–902. 19581375 10.1189/jlb.0209050 PMC2791992

[42] Bergman P, Walter-Jallow L, Broliden K, Agerberth B, Soderlund J (2007) The Antimicrobial Peptide LL-37 Inhibits HIV-1 Replication. Curr HIV Res 5: 410–415. 17627504 10.2174/157016207781023947

[43] Wong JH, Legowska A, Rolka K, Ng TB, Hui M, et al (2011) Effects of cathelicidin and its fragments on three key enzymes of HIV-1. Peptides 32: 1117–1122. 21539873 10.1016/j.peptides.2011.04.017

[44] Gordon YJ, Huang LC, Romanowski EG, Yates KA, Proske RJ, et al (2005) Human Cathelicidin (LL-37), a Multifunctional Peptide, is Expressed by Ocular Surface Epithelia and has Potent Antibacterial and Antiviral Activity. Curr Eye Res 30: 385–394. 16020269 10.1080/02713680590934111 PMC1497871

[45] Kota S, Sabbah A, Chang TH, Harnack R, Xiang Y, et al (2008) Role of human beta-defensin-2 during tumor necrosis factor-alpha/NF-kappaB-mediated innate antiviral response against human respiratory syncytial virus. J Biol Chem 283: 22417–22429. 18567888 10.1074/jbc.M710415200 PMC2504899

[46] Mookherjee N, Brown KL, Bowdish DME, Doria S, Falsafi R, et al (2006) Modulation of the TLR-Mediated Inflammatory Response by the Endogenous Human Host Defense Peptide LL-37. J Immunol 176: 2455–2464. 16456005 10.4049/jimmunol.176.4.2455

[47] Lai Y, Adhikarakunnathu S, Bhardwaj K, Ranjith-Kumar CT, Wen Y, et al (2011) LL37 and Cationic Peptides Enhance TLR3 Signaling by Viral Double-stranded RNAs. PLoS One 6: e26632. 22039520 10.1371/journal.pone.0026632 PMC3198786

[48] Singh D, Qi R, Jordan JL, San Mateo L, Kao CC (2013) The human antimicrobial peptide LL-37, but not the mouse ortholog, mCRAMP, can stimulate signaling by poly(I:C) through a FPRL1-dependent pathway. J Biol Chem 12: 8258–8268. 10.1074/jbc.M112.440883 PMC3605644 23386607

